# Extracellular Vesicles: A Possible Link between HIV and Alzheimer’s Disease-Like Pathology in HIV Subjects?

**DOI:** 10.3390/cells8090968

**Published:** 2019-08-24

**Authors:** Sunitha Kodidela, Kelli Gerth, Sanjana Haque, Yuqing Gong, Saifudeen Ismael, Ajay Singh, Ishrat Tauheed, Santosh Kumar

**Affiliations:** 1 Department of Pharmaceutical Sciences, University of Tennessee Health Science Center, 881 Madison Ave, Memphis, TN 38163, USA; 2 Department of Anatomy and Neurobiology, The University of Tennessee Health Science Center, 855 Monroe Avenue #515, Memphis, TN 38163, USA; 3 Department of Pediatric Pulmonology, Le Bonheur Children Hospital, 50 N. Dunlap st, Memphis, TN 38103, USA

**Keywords:** HIV, exosomes, extracellular vesicles (EVs), beta-amyloid, Alzheimer’s disease, neurodegeneration, HAND, HIV-TAT

## Abstract

The longevity of people with HIV/AIDS has been prolonged with the use of antiretroviral therapy (ART). The age-related complications, especially cognitive deficits, rise as HIV patients live longer. Deposition of beta-amyloid (Aβ), a hallmark of Alzheimer’s disease (AD), has been observed in subjects with HIV-associated neurocognitive disorders (HAND). Various mechanisms such as neuroinflammation induced by HIV proteins (e.g., Tat, gp120, Nef), excitotoxicity, oxidative stress, and the use of ART contribute to the deposition of Aβ, leading to dementia. However, progressive dementia in older subjects with HIV might be due to HAND, AD, or both. Recently, extracellular vesicles (EVs)/exosomes, have gained recognition for their importance in understanding the pathology of both HAND and AD. EVs can serve as a possible link between HIV and AD, due to their ability to package and transport the toxic proteins implicated in both AD and HIV (Aβ/tau and gp120/tat, respectively). Given that Aß is also elevated in neuron-derived exosomes isolated from the plasma of HIV patients, it is reasonable to suggest that neuron-to-neuron exosomal transport of Aβ and tau also contributes to AD-like pathology in HIV-infected subjects. Therefore, exploring exosomal contents is likely to help distinguish HAND from AD. However, future prospective clinical studies need to be conducted to compare the exosomal contents in the plasma of HIV subjects with and without HAND as well as those with and without AD. This would help to find new markers and develop new treatment strategies to treat AD in HIV-positive subjects. This review presents comprehensive literatures on the mechanisms contributing to Aβ deposition in HIV-infected cells, the role of EVs in the propagation of Aβ in AD, the possible role of EVs in HIV-induced AD-like pathology, and finally, possible therapeutic targets or molecules to treat HIV subjects with AD.

## 1. Introduction

According to the latest data from the United Nations Programme on HIV/AIDS (UNAIDS), there were approximately 36.9 million people living with HIV/AIDS globally in 2017 [1]. Since the introduction of antiretroviral therapy (ART), HIV/AIDS-related mortality has been drastically reduced. Consequently, the longevity of HIV-infected subjects has been significantly prolonged [2]. In 2016, 47% of people living with HIV or AIDS in the United States were aged 55 or older [3]. As they live longer, the prevalence of age-related comorbidities, neurocognitive disorders, cardiovascular diseases, and cancer increase in the HIV-infected population. Despite successful suppression of plasma viral load, the symptoms of HIV-associated neurocognitive disorders (HAND) continue to be observed in clinics [4,5]. Further, HIV infection can induce premature aging despite ART treatment [6] and can lead to neurocognitive decline similar to that seen in patients with Alzheimer’s disease [7] (AD). Alzheimer’s disease (AD) is the most common age-related neurodegenerative disease. It is characterized by progressive memory loss and declining cognitive function and is the fifth leading cause of death in people over the age of 65 [8]. According to the latest Alzheimer’s Association report, one in 10 people of age 65 and older has AD and its prevalence increases with age [9]. AD involves irreversible neuronal degeneration, which gives rise to multiple clinically detectable neurological impairments, which dramatically affect the quality of life [10,11]. Multiple pathogenic hypotheses have been put forward about the pathogenesis of AD including extracellular beta-amyloid (Aβ) peptide deposition, intracellular accumulation of hyperphosphorylated tau, cholinergic dysfunction, inflammation and oxidative stress [12]. Accumulation of Aβ in the brain occurs with aging and is an important pathological event in AD. Interestingly, a higher accumulation of Aβ has been observed in HIV-positive subjects compared to uninfected people [13,14]. Case reports also suggest the development of AD in HIV-infected subjects [15]. Recently, Turner et al. reported in a case study that progressive dementia in older subjects with HIV might be due to HAND, AD, or both. Therefore, it is critical to understand the mechanisms of neurodegenerative processes in HIV positive subjects to develop treatment strategies for this subpopulation. Recently, EVs (mainly exosomes) have gained recognition for their importance in understanding the pathology of HIV-induced neurodegenerative disorders, as they have been reported to package the HIV viral proteins Tat, gp-120, and Nef, which can induce neurotoxicity [15,16,17,18,19]. Moreover, EVs play a major role in HIV replication and its associated complications due to their overlapping use of biogenesis pathways [20,21]. Interestingly, colocalization of exosomes with Aβ has been observed in cases of AD [22,23]. Further, Pulliam et al. reported that plasma neuron derived exosomes (NDEs) can serve as biomarkers of cognitive impairment in HIV subjects and AD patients and could also be used to distinguish between HIV-associated dementia and AD in HIV-infected subjects [22,24]. Thus, studying the role of EVs in neurodegeneration may impact our understanding and eventual treatment of HAND and AD in HIV subjects. In this review, we briefly discuss the mechanisms contributing to amyloid beta deposition (a hallmark of AD) in HIV infection, the role of EVs in propagating AD, and a proposed role for EVs in developing AD in HIV subjects. 

### 1.1. Mechanisms Contributing to Aβ Deposition in HIV-Infected subjects

Various mechanisms involved in the HIV infection of macrophages, microglia, and astrocytes may play a robust role in neuronal injury and in the interruption of normal neurological mechanisms, contributing to HIV-associated dementia or AD. The neuronal injury could result from overlapping mechanisms involving viral proteins released from infected cells and inflammatory processes associated with HIV infection. Alterations in the levels of β-amyloid, damage to the blood-brain barrier (BBB), and even ART could potentiate the development of AD-like pathology in the HIV positive population. 

Plaques composed of Aβ aggregates and tau-containing neurofibrillary tangles, both hallmarks of AD, have been found within the brain tissue of those affected by AD and in HIV patients [10,25,26]. However, characteristics of these plaques differ between the two disease states. HIV-infected individuals have an abundance of Aβ plaques that are located both perivascularly and within neurons, differing from typical AD plaques that are mostly parenchymal and extracellular [26,27]. Furthermore, they have been classified as diffuse in nature, a morphology that is consistent with the early stages of Aβ plaque development [28] and similar to plaques commonly found in cognitively intact older adults, generally precluding an AD diagnosis [29]. Moreover, variations in Aβ and tau biomarkers from cerebrospinal fluid between patients with HAND and AD have led some to suggest that mechanisms of neuronal injury may differ [30]. In addition to differences in nature of AB plaques and Tau pathology, several differences exist between HAND and AD [31]. Selective cortical neurodegeneration with deficits in primary sensory, motor and premotor cortices occur in HIV subjects [32], whereas in subjects with AD, the medial temporal, limbic, and association cortices are affected first, and primary sensorimotor and visual cortices only later [33]. Memory deficits often reflect learning inefficiency rather than amnestic disorder in HAND subjects [34,35]. Visuospatial abilities are intact in HAND subjects [36], whereas in AD patients these abilities are compromised [37]. Similarly, semantic memory that supports language functioning is retained in HAND subjects [38], but it is deteriorated in AD patients [37].

Assessment of spinal fluid biomarkers and position emission tomography imaging seems to be a promising method to distinguish between HAND and AD [31,39,40]. Here, we describe the mechanisms contributing to Aβ deposition in HIV-infected patients (Table 1).

#### 1.1.1. Neuroinflammation

HIV-infected macrophages and monocytes can cross the BBB and induce neuroinflammation, providing one possible mechanism for the pathogenesis of HAND [41,42]. It is likely that inflammatory processes associated with HIV infection alter certain pathways and proteins known to be involved in AD, such as Aβ and tau production and stress-related pathways in the brain [43]

Aβ production results from a pathological cleavage of the amyloid precursor protein (APP) by β-secretase (BACE-1), which yields the C99 fragment; when C99 undergoes a second cleavage by γ-secretase, Aβ is released into the extracellular medium, where it may form aggregates [44,45,46,47].

Aβ plaques and tau-containing neurofibrillary tangles are central to the combined neuroinflammatory-amyloid hypothesis of AD pathophysiology, characterized by a dysregulated immune response following an initial inflammatory stimulus, such as pathogenic infection. Following the initial inflammatory stimulus, resident CNS microglia secrete pro-inflammatory cytokines (IL-1β, IL-6, TNFα) and chemokines (CCL2, CCR3, CCR5), which recruit additional microglia and astrocytes to the inflammatory site. Although this process is controlled under normal circumstances, excessive Aβ production and tau hyper-phosphorylation dysregulate the immune clearance mechanism, potentiating a neuroinflammatory cycle of excessive cytokine and chemokine production and triggering further cleavage of APP [48].

The neuroinflammatory environment is associated with cholinergic neuron degeneration; synaptic deficits; and an increase in oxidative stress, apoptosis, and autophagy, eventually leading to cognitive and behavioral deficits [49]. In the case of HIV infection, the neuroinflammatory cycle persists because the CNS serves as a viral reservoir wherein HIV-infected microglia and macrophages secrete viral proteins like Tat [26,50]. Tat is known to disrupt the integrity of the BBB by altering the expression and distribution of tight junction proteins. Moreover, Aβ and Tat may act synergistically in brain microvascular endothelial cells (BMVECs) at the BBB, enhancing the expression of pro-inflammatory genes via the NF-κβ pathway [51].

#### 1.1.2. HIV Proteins Gag, GP-120, and TAT

##### Tat and Aβ

The HIV viral protein Tat, released from HIV-infected macrophages and glial cells, is a key activator for HIV transcription [26]. Tat is produced from proviral DNA in brain reservoirs of HIV patients and is detectable even in the cerebrospinal fluid of patients on ART [52]. Tat interacts with Aβ molecules both directly and indirectly. Hategan et al. observed that Tat forms extracellular complexes with Aβ in vitro and in vivo, and that these complexes were more neurotoxic than Aβ alone [53]. Further, Tat has been shown to inhibit the proteolytic functions of neprilysin, a key enzyme responsible for Aβ degradation in the brain [54,55] and to inhibit the microglial phagocytosis of Aβ [56]. Tat may also contribute to Aβ production, deposition, and an AD-like pathology through several other mechanisms. 

Tat increases extracellular Aβ by inducing the release of Aβ 1–42 (a major component of extracellular plaques) and promoting the accumulation of cell-bound Aβ aggregates [57]. Kim et al. demonstrated that, in vitro, Tat enhanced β-secretase-mediated cleavage of APP, resulting in a 5.5 times increased expression of Aβ 1–42. Further, Tat injection in mice also increased Aβ 1–42 and was associated with an increase in the number and size of Aβ plaques [58]. 

It has been suggested that intracellular Aβ clearance may be impaired by Tat, as increased intraneuronal Aβ has been observed in HIV-infected patients [27]. Additionally, Tat binds to low-density lipoprotein receptor-related protein (LRP) expressed on neurons, which promotes neuronal uptake of Tat and prevents LRP from degrading its other ligands, including APP and Aβ [59]. Once inside neurons, Tat may alter Aβ metabolism by disturbing endolysosome function [26]. Within endolysosomes, Tat causes the accumulation of β-secretase, APP, and Aβ, which enhances β-secretase-mediated cleavage of APP [26].

In addition to altering the expression of endothelial tight junctions and increasing BBB permeability, Tat also impairs Aβ clearance from the brain by disturbing the expression of other BBB proteins involved in Aβ translocation, such as LRP-1 and RAGE (receptor for advanced glycation end products) [26,60]. Specifically, LRP-1 transports Aβ from the brain to the blood and hence facilitates its clearance, while RAGE promotes its uptake into the brain. Chen et al. demonstrated a decrease in brain endothelial LRP-1 expression following Tat exposure, with a corresponding upregulation of RAGE [60]. Furthermore, Jiang et al. observed a Tat-induced increase in Aβ translocation across brain endothelial cells, along with increased reactive metabolite production via the Ras signaling pathway [61].

##### Gp-120 and Aβ

Similar to Tat, the viral envelope protein gp120 may also contribute to Aβ production by altering the trafficking of Aβ, promoting accumulation and β-secretase-mediated cleavage of APP [62,63]. Gp120 has also been shown to stimulate Aβ 1–42 secretion in primary rat fetal hippocampal cultures [57]. 

##### Gag and Aβ

HIV Gag proteins are essential to perpetuating the life cycle of the virus, playing important roles in viral assembly and maturation [64]. Chai et al. have recently elucidated a possible link between HIV-Gag and Aβ production by demonstrating that macrophages and microglia have a high APP production rate that is likely involved in blocking HIV viral replication. APP within macrophages and microglia binds and sequesters Gag in lipid rafts to prevent viral production and spreading. As a defense mechanism, HIV-Gag increases APP breakdown by secretases, contributing to the formation of β-amyloid [65].

#### 1.1.3. Excitotoxicity and Oxidative Stress

Glutamate, a nonessential amino acid, is the primary excitatory neurotransmitter in the CNS [66]. It exerts its signaling effect when it is released from neural cells into the extracellular space and binds to N-methyl-aspartate receptors (NMDARs), resulting in intracellular Ca^2+^ influx, subsequent action potentials, and neuronal excitation [67]. After a signaling event, excitatory amino acid transporters, located on astrocytes and other glial cells, remove excess glutamate from synaptic clefts to maintain homeostatic conditions. However, in the case of neurodegenerative diseases, such as dementia, AD, or HAND, glutamate uptake can become impaired [68]. Excess glutamate in synaptic clefts results in excitotoxicity and neuronal damage, and disruptions in glutamate regulation have been correlated with cognitive impairment in patients with HIV [41].

In HIV-associated neurotoxicity, glutamate homeostasis is dysregulated when viral proteins such as Tat and gp120 cause infected astrocytes, microglia, and macrophages to release cytokines and other pro-inflammatory chemicals that activate NMDARs and cause oxidative stress [69]. The ensuing inflammatory process prompts more presynaptic glutamate release while also downregulating excitatory amino acid transporter gene expression and reducing postsynaptic glutamate uptake [68]. The mild but sustained increase in extracellular glutamate seen in HIV infection further activates NMDARs, increasing intracellular calcium levels and leading to apoptosis [70,71]. In addition, Tat produced from HIV-infected astrocytes has been shown to upregulate nuclear factor erythroid 2-related factor 2 activity—an antioxidant pathway that promotes excitotoxicity downstream via glutamate-cysteine transport [72]. 

Oxidative stress-mediated neurotoxicity in HIV patients is thought to be a result of both direct neuronal injury by HIV viral proteins (gp120, Tat, Nef, Vpr) and indirect injury from infected macrophages/microglia and astrocytes [71]. 

The viral proteins Tat and gp120 directly induce neuronal apoptosis via disruptions in calcium homeostasis, caspase activation, and reactive metabolite accumulation [73,74,75]. Similarly, when neurotoxins and viral proteins are released from HIV-infected macrophages/microglia and astrocytes into the extracellular space, excitotoxicity causes free radical production following NMDAR activation and sustained elevations in intracellular Ca^2+^ levels [71,76]. Further, Saribas et al. demonstrated increased oxidative stress, as evidenced by decreased intracellular glutathione levels, when astrocytic EVs carrying Nef were introduced to neuronal cells [16]. Another study by Ferrucci et al. revealed a reduction in intracellular glutathione when astrocytes were exposed to the viral protein Vpr. Importantly, Vpr is found in high concentrations in the serum and cerebrospinal fluid of patients with late-stage HIV, and is associated with neurological complications [77].

Furthermore, Tat has been implicated in BBB disruption via oxidative stress pathways. Tat released from infected brain-derived macrophages has been shown to increase oxidative stress and reduce glutathione levels in neurons and brain endothelial cells [78]. 

#### 1.1.4. BBB Damage

The BBB serves as a protective wall around the brain, primarily consisting of brain microvascular endothelial cells (BMVECs) and astrocytes, as well as pericytes [79]. Together with microglia, immune regulators, these cells maintain homeostasis under healthy conditions. However, homeostasis is disrupted during an assault or infection, such as HIV invasion, leading to the activation of select cells in the CNS. 

HAND and AD are characterized by a disrupted BBB [80,81]. HIV enters into the brain in the early stages of infection and resides within microglial cells, known viral reservoirs, and also infects astrocytes and endothelial cells [82]. HIV crosses the BBB by hiding within the peripheral monocytes, which continuously enter the brain and replace resident macrophages [83]. Once inside, the HIV-infected and immune-activated macrophages release HIV gene products, inflammatory cytokines, and brain endothelial adhesion molecules [84]. 

One of the consequences of these events is damage to human BMVE cells inducing oxidative stress, which leads to an impaired BBB. Simultaneously, the adhesion molecules attract more infected macrophages, which infiltrate easily through the damaged BBB. Additionally, HIV proteins such Tat, gp120, and Nef directly damage the BBB by altering the levels of tight junction proteins, nitric oxide, pro-inflammatory and interferon-inducible genes, leukocyte adhesion, trans-endothelial electrical resistance, and matrix metalloproteinases, leading to increased permeability of brain endothelial cells [85,86,87,88,89,90,91].

Aging also increases BBB permeability [92], thus, as with the general population, BBB integrity is compromised in aging HIV patients. Similarly, the likelihood of developing AD increases as HIV patients age. The aging brain itself generates β-amyloid, and the influx of circulating Aβ into the brain by specific resident receptors could be a potential source for deposition of brain Aβ [93]. With a damaged BBB, Aβ transport may be even higher, resulting in greater insult to the brain microenvironment. 

#### 1.1.5. Antiretroviral Therapy (ART)

As mentioned previously, ART is used to control HIV but can also contribute to AD by inducing oxidative stress. Apart from that, patients on ART demonstrate immune reconstitution syndrome, which comprises of vasculitis, hyperlipidemia, diabetes, and coronary artery disease, common risk factors for AD [94]. A study on nelfinavir, a commonly prescribed HIV protease inhibitor, found that it inhibits an insulin degrading enzyme that also degrades Aβ, thus increasing the risk of AD [95,96].

Considering the mechanisms of developing HIV-associated AD, there remains a missing link between HIV in the periphery and its invasion of the CNS. Although a compromised BBB could play a role, HIV may enter the brain prior to the reduction in BBB integrity. Macrophages are known reservoirs for viral particles, however EVs have also recently been considered as potential carriers. 

## 2. EVs, HIV, and AD 

EVs/exosomes are known to transport a variety of biologically important molecules, such as lipids, carbohydrates, proteins, mRNAs, miRNAs, small DNA molecules, etc. [99,100]. EVs are biological nanoparticles (<200 nm), released and taken up by almost all kinds of cells [101,102,103,104,105]. Exosomesoriginate within the endosomal system as intraluminal vesicles contained within multivesicular bodies derived from the early endosome [106,107]. During formation, EVs can package molecular cargo from the cytoplasm. EV cargo varies widely depending upon the parent cell and cellular environment. Studies have shown the presence of specific lipid sequence motifs that can generate signals and sort molecules to be loaded into EVs; however, the exact sorting mechanism is not fully understood [106]. After being released from cells, EVs adhere to recipient cells and are commonly internalized either via fusion or endocytosis. Fusion causes direct release of exosomal content into the cytoplasm, whereas endocytosis might direct components to the endosomal-degradation pathway [103]. Either way, exosomal cargo ultimately causes a cellular response specific to the nature of the substances being delivered. EVs’ biology and their role in HIV and neurodegenerative disorders have been described in other reviews [17,19,21]. 

HIV has been demonstrated to hijack the mechanistic pathway of exosome formation and release, i.e., intraluminal vesicle formation and budding. Both HIV-infected macrophage-derived EVs and HIV virions are shown to carry a similar set of host proteins, and they are also very similar in size, which may indicate that EVs and HIV have similar origins [17,107]. Moreover, EVs are shown to carry viral proteins like Tat, Nef, and gp120 [19,108,109]. In fact, components of the endosomal sorting complex required for transport (ESCRT) machinery, important regulators of exosomal formation, cargo loading and vesicle release, are shown to interact with HIV [18]. HIV also induces differential packaging of specific contents within EVs in terms of mRNAs, miRNAs, proteins, cytokines etc. [100,110,111]. Furthermore, EVs can package Aβ, APP, and tau proteins, which contribute to HAND [23,112].

In addition to their role in intracellular communication, EVs/exosomes can be used as biomarkers for specific conditions. NDEs from HIV-infected and non-infected subjects showed that neuropsychological impairment decreases the total quantity of NDEs and enriches them with high-mobility group box 1 (HMGB1), neurofilament-light (NF-L), and Aβ. These observations suggest the potential application of NDEs as biomarkers of cognitive impairment in HIV patients [24,113]. Again, the number of monocyte-derived exosomes has been shown to be higher in HIV-infected subjects, and they effectively facilitate the viral infection [114]. Exosomes are already being studied as prospective carriers of drug delivery vehicles, e.g., catalase-loaded exosomes have been shown to be neuroprotective both in vitro and in in vivo Parkinson’s disease models [115]. 

Role of EVs in Propagating Alzheimer’s Disease

Epidemiologic studies have demonstrated that myriad risk factors, both genetic and non-genetic, contribute to the development and progression of AD [11]. Recently, EVs/exosomes have been shown to play a role in propagation of AD pathology [23]. The major physiological roles of EVs/exosomes include clearance of cellular waste, modulation of immune responses, and intracellular communication [116,117]. EVs also participate in regulating neuronal development, regeneration, and modulation of synaptic functions [118]. 

In the amyloidogenic pathway, APP is processed by β-secretase in early endosomes and accumulates in multivesicular bodies. It is then released into the extracellular space through EVs/exosomes, suggesting a contributory role in extracellular Aβ accumulation [119,120]. Exosomal reduction is associated with improved brain amyloid clearance in a 5×FAD mouse model of familial AD [121]. Goetzl et al. reported that Aβ is packaged in astrocyte exosomes as well as in NDEs collected from the plasma of AD patients [122]. Similarly, Sinha et al. demonstrated that AD brain-derived exosomes contain increased amounts of oligomeric Aβ, and that those exosomes may be responsible for the neuron-to-neuron transfer of the toxic peptide. They also suggested that exosomes are co-localized in the neurons of AD patients, suggesting its role in Aβ sorting and oligomerization [23]. EVs/Exosomes also play significant role in the clearance of Aβ [123]. Intracerebral administration of neuron-derived exosomes into APP transgenic mice resulted in decreased levels of Aβ, accumulation of amyloid and Aβ mediated synaptotoxicity in the hippocampus. Further, neuronal exosomes which are rich in glycophingolipids, membrane glycolipids, could bind with Aβ both in vitro and in vivo and induce their clearance by microglia, thereby promoting plaque clearance in APP transgenic mice [124,125]. Therefore, targeting Aβ by the administration of neuronal exosomes may open a novel therapeutic avenue.

Another hallmark of AD is intracellular accumulation of neurofibrillary tangles composed of hyperphosphorylated tau, a microtubule-associated protein with two isoforms, 3R and 4R [126]. Abnormal isoform distribution impairs microtubule stabilization and material transport. Usually the spread of hyperphosphorylated tau occurs after the formation of amyloid plaques in the hippocampus and later spreads to the temporal cortex [123]. Exosomes mediate the distribution of tau protein in different parts of the brain, resulting in cognitive decline [127]. Additionally, exosomes derived from the brains of AD patients have increased levels of phosphorylated tau protein in comparison with healthy controls [128]. 

EVs/Exosomes also play a significant role in mediating neuroinflammation in the AD brain, as they carry inflammatory mediators and exchange them between neurons and glia [129] potentiating inflammatory cascades. As EVs package and transport toxic proteins implicated in both AD and HIV (Aβ/tau and gp120/Tat, respectively) and given that Aβ is also elevated in plasma NDEs isolated from HIV patients, it may be reasonable to suggest that neuron-to-neuron exosomal transport of Aß and tau also contributes to AD-like pathology in HIV-positive subjects.

Role of EVs in Developing AD-Like Pathology in HIV Subjects

The HIV-1 viral proteins gp120 and Tat have been found to be packaged in exosomes [108,129]. Both Tat and gp120 have demonstrated neurotoxicity in vitro and in vivo. Furthermore, Tat seems to work synergistically with gp120 and glutamate to potentiate cell death [68]. Additionally, peripheral EVs containing these viral proteins may disrupt the BBB and hence facilitate transport of these neurotoxins into the CNS and contribute to the development of HAND [18]. Importantly, Rahimian et al. have demonstrated that Tat packaged in exosomes from HIV-1-infected astrocytes is biologically active [19]. 

Further, Sinha et al. have demonstrated that EVs/exosomes may play a major role in neuron-to-neuron transportation of the toxic proteins amyloid beta (Aβ) and tau, contributing to the pathogenesis of AD [23]. In addition to being implicated in AD pathophysiology, Aβ has been shown to be elevated in both the brains of HIV-infected patients and in Tat-exposed neuronal cells in vitro [24]. Further, the presence of HIV Tat has an inhibitory effect on neprilysin, an enzyme that degrades Aβ peptides [130]. Additionally, Tat has been shown to stimulate the release and cleavage of Aβ precursors [26,57,58], promote Aβ accumulation [57,58], alter BBB permeability [26,60], disrupt normal Aβ trafficking and clearance [27,61], and alter Aβ metabolism [26,56,59], providing several possible mechanisms of neuronal Aβ accumulation in patients with HIV. 

**Figure 1:**
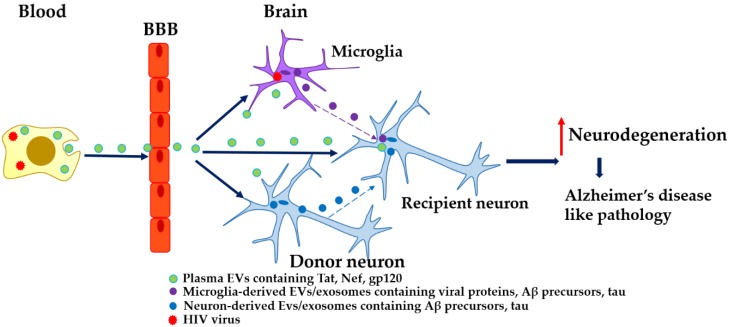
Possible mechanisms of EVs/exosome in propagating HIV-induced Alzheimer’s disease- like pathology.

Therefore, it is likely that the development of HIV-associated dementia may involve EV/exosomal transport of viral proteins from the periphery and across the BBB, where they are taken up by cells in the CNS. EVs are also involved in transport of toxic proteins from glial cells to neurons. These neurotoxins contribute to the increase in neuronal Aβ deposition and potentiate neuronal cell death, (Figure 1). Since EVs package and transport toxic proteins implicated in both AD and HAND and given that Aβ is also elevated in plasma NDEs isolated from HIV patients (Table 2), it is reasonable to suggest that neuron-to-neuron exosomal transport of Aβ and tau also contributes to the ongoing pathology of cognitive dysfunction in HIV-infected subjects. Further, as previously mentioned, exosomal Tat released from HIV-infected astrocytes is biologically active. Therefore, it is likely that exosomal viral proteins also contribute to Aβ accumulation in patients with HIV.

## 3. Interventions to Target Aβ Mediated via EVs

Growing evidence suggest that EVs/exosomes are involved in transport of Aβ across cells under HIV infection. Thus, targeting EV Aβ in HIV-infected cells may mitigate the neurodegeneration. Recently, caffeine has been shown to inhibit Tat-induced Aβ production and tau phosphorylation [132], and epidemiological studies suggest that caffeine can decrease the risk of dementia/AD [133]. Therefore, caffeine can be used to target both cellular and EVAβ and tau to treat AD in HIV subjects. Since autophagy is altered in both HAND and AD, drugs which can modulate autophagy could be useful in treating both conditions. Rapamycin can affect autophagy and has shown promising results in improving AD symptoms in preclinical models [134]; however, it has poor BBB penetration. Thus, loading rapamycin in EVs may overcome this limitation due to their ability to cross the BBB [135,136]. Moreover, EVs have been investigated as drug delivery vehicles in various disease conditions including neurodegenerative disorders [115,137,138]. Recently sunitinib, an anticancer drug, has been shown to promote Tat-mediated autophagy and decrease neurodegeneration [139]. However, the sunitinib level in the brain is affected by the drug efflux proteins P-glycoprotein (P-gp) and breast cancer protein (ABCG2) [140]. Interestingly, exosomes have been shown to play a role in developing drug resistance by transferring P-gp [141,142]. Therefore, targeting exosomal P-gp could improve drug levels in the brain to further promote autophagy. Curcumin can be a potential compound to treat Tat-induced Aβ deposition because it degrades Tat protein [143]. Further, it has been shown to be effective against various neurodegenerative disorders, including AD [144,145], and its neuroprotective properties have previously led to testing of the compound in phase 1 clinical trials. However, curcumin could not move forward to other phases of clinical trials due to its pharmacokinetic limitations, i.e., poor bioavailability/absorption and rapid metabolism/elimination. Recently, curcumin-loaded exosomes have been shown to overcome these limitations, which can facilitate its neuroprotective properties [146,147,148]. Activation of NOD-like receptor protein 3 (NLRP3) leads to neuroinflammation by releasing cytokines and other factors contributing to the development of AD [149,150]. Recently, HIV-Tat has been found to prime and activate microglial NLRP3 inflammasome, suggesting NLRP3 as a therapeutic target to ameliorate Tat-mediated neuroinflammation [151]. Interestingly, NLRP3 activation is associated with enhanced extracellular vesicle secretion, which has been suggested to play a role in NLRP3-mediated IL-1β release [152] and its role in AD pathology. Therefore, targeting NLRP3 may be helpful for ameliorating neuroinflammation in subjects with HIV.

Despite successful suppression of plasma HIV RNA with the use of ART, the virus still persists in the infected glial cells of CNS due to an inability of ARVs to cross the BBB. The EVs released from infected glial cells can transfer viral proteins to neurons leading to their inflammation and/degeneration with similarities to AD. However, differentiating HAND from AD in aging HIV subjects is a major issue for the geriatric population due to the commonalities between these two disorders [31,43]. However, in addition to differences in nature of Aβ plaques and Tau pathology, several differences exist between HAND and AD [31]. Assessment of spinal fluid biomarkers and position emission tomography imaging seems to be a promising method to distinguish between HAND and AD [31,39,40]. However, the former procedure is painful, whereas the latter is expensive and not widely available in clinics. Therefore, there is a need to develop a stable, feasible, reliable, and costeffective marker to distinguish between these two conditions in HIV infected subjects. EVs may serve this purpose, as they package different biomolecules based on the type of stimulus or disease. Pulliam et al. recently showed that Aβ levels did not vary between NDEs of HIV subjects with and without cognitive impairment [153] whereas these levels were reported to be high in NDEs of AD patients compared to controls [128], suggesting NDEs may serve as markers to distinguish between HAND and AD. Furthermore, profiling of exosomal cargo in HIV and AD subjects could provide insights in our understanding and eventual treatment of HAND and AD. 

## 4. Conclusions

HIV infection causes premature aging and leads to the development of age-related complications, in particular, neurodegenerative disorders such as AD. Deposition of Aβ has been observed in patients with HAND, and differentiating between HAND and AD in the geriatric population is becoming a major challenge to clinicians. EVs can serve as a possible link between HIV and AD, as they package and transport toxic proteins implicated in both conditions (gp120/Tat and Aβ/tau, respectively) between CNS cells, leading to neurodegeneration. However, clinical prospective studies need to be conducted to confirm the role of EVs as markers to distinguish between HAND and AD in HIV subjects and to develop new treatments for these neurodegenerative conditions. Further, EVs themselves could be used as therapeutic modalities or therapeutic targets in this subpopulation.

## Figures and Tables

**Table 1:** Summary of HIV-induced mechanisms contributing to neurodegeneration/AD-like pathology.

HIV Components	Mechanism	Consequences/Conclusion	References
**Neuro-Inflammation**			
HIV-infected microglia, macrophages, astrocytes	HIV infection of CNS cells provides an inflammatory stimulus and promotes secretion of viral proteins, e.g., Tat, gp-120	Cycle of excessive cytokine/chemokine production, Aβ production, ROS production	[48,97]
**HIV Proteins**			
Tat	Forms highly neurotoxic complexes with Aβ; inhibits neprilysin; inhibits microglial phagocytosis of Aβ; stimulates Aβ 1–42 release and promotes plaque accumulation; enhances cleavage of Aβ precursors; alters BBB permeability (see BBB damage)	More Aβ is produced in the CNS while less is cleared; alteration of Aβ degradation/metabolism; BBB damage	[27,53,54,55,57,59,60]
Gp-120	Like Tat, alters Aβ trafficking/accumulation and enhances cleavage of Aβ precursors; alters BBB permeability (see BBB damage)	More Aβ is produced in the CNS while less is cleared	[62,63]
Gag	Aβ precursor, APP, binds and sequesters Gag in lipid rafts within macrophages to prevent viral spreading. In defense, Gag enhances APP cleavage	Increased Aβ production	[65]
**Excitotoxicity and oxidative stress**			
HIV-infected microglia, macrophages, astrocytes	Infected CNS cells release pro-inflammatory chemicals that activate NMDARs	Excessive activation of NMDARs promotes excitotoxicity and free radical production	[69,70,71,76]
Tat, gp-120, Nef, Vpr	Viral proteins injure neuronal cells directly and disrupt calcium homeostasis, activate caspases, promote ROS accumulation; alter BBB permeability via oxidative stress pathways (see BBB damage)	Excitotoxicity/Induction of oxidative stress; BBB damage	[73,74,75,77]
**BBB Damage**			
HIV virus	Affect HBMECs by releasing HIV gene products, inflammatory cytokines, and adhesion molecules on brain endothelium	Induction of oxidative stress	[84]
Tat, gp120, Nef	Alteration of the levels of tight junction proteins, nitric oxide, pro-inflammatory and interferon-inducible genes, leukocyte adhesion, trans-endothelial electrical resistance, and matrix metalloproteinases	Increased permeability of HBMEC	[58,85,87,88,89,90]
Aging	β-amyloid generation	Higher Aβ	[98]
**ART Medication**			
ART	Increased oxidative stress; IRIS promotes vasculitis, hyperlipidemia, diabetes, coronary artery disease; Nelfinavir inhibits Aβ degradation enzyme	Contributes to AD risk factors and Aβ accumulation	[94,95,96]

**Table 2:** HIV elements and Aβ packaged within EVs/exosomes of CNS cells.

HIV Proteins	Source	Reference	AD	Source	Reference
Tat	Astrocytes	[19]	Aβ	Primary cortical neurons	[131]
				Cerebrospinal fluid	[131]
				Neuroblastoma cell lines (SH-SY5Y)	[23]
Gag	Monocyte-derived macrophages	[114]		Brain tissues from AD patients	[23]
				Astrocyte derived exosomes from plasma of AD patients	[122]
				NDEs from plasma of AD patients	[122]
